# Seins surnuméraires axillaires bilatéraux

**DOI:** 10.11604/pamj.2014.17.45.3203

**Published:** 2014-01-22

**Authors:** Nadia Khoummane, Mounia Yousfi

**Affiliations:** 1 Service de Gynécologie Obstétrique, de Cancérologie et de Grossesses à haut risque, Maternité Souissi de Rabat, Maroc

**Keywords:** Seins surnuméraires, ligne axillaire, glandes mammaires, supernumerary breasts, axillary line, mammary gland

## Image en medicine

Au cours du dévelopement embryologique, les seins appraissents sur deux crètes mammaires initiales se situant sur une ligne se prolongeant de la ligne axillaire à la face antéro-interne de la cuisse. Les seins surnuméraires ( polymastie ou encore hypermastie) sont dues à l'absence de régression des bourgeons mammaires au cours de la vie embryonnaire. Les glandes mammaires accesoires axillaires sont fréquentes mais le plus souvent confondues avec le prolongement axillaire du sein. Leur diagnostic reste difficile en l'absence de mamelon et d'engorgement mammaire pendant l'allaitement et la gestation, ceci explique la confusion fréquente de cette anomalie avec les lipomes et adénopathie axillaire. Nous rapportons ici le cas d'une patiente de 36 ans, célibataire, G0P0, qui se présentait pour masse axillaire bilatérale depuis trois ans augmentant progressivement de volume, ces dernières était molles bien limitées non douleureuse à la plaplation, mobile par rapport au plan profond et adhérente au plan superficiel sans signes inflammatoires en regard mesurant environ 6cm /6cm faisant évoquer en premier lieu un lipome axillaire, une échographie faite a révèlé une structure mammaire ectopique. Le traitement a conssisté en une exérèse chirurgicale bilatérale avec examen anatomopathologique confirmant le diagnostic de seins surnuméraires sans lésion tumorale décelable.

**Figure 1:**
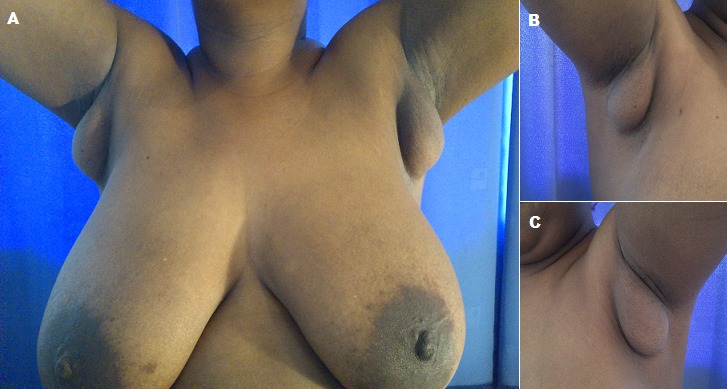
A) seins surnuméraires axillaires bilatéraux; B) sein surnuméraire droit sans mamelon portant confusion avec un lipome axillaire; C) masse axillaire gauche correspondant à un sein accessoire

